# Nanocrystals of a new camptothecin derivative WCN-21 enhance its solubility and efficacy

**DOI:** 10.18632/oncotarget.16159

**Published:** 2017-03-13

**Authors:** Jia You, Yuyuan Chen, Zubaeda M. Mohamed Alsayeh, Xingyu Shen, Chun Li, Pengxuan Zhao, Fei Chen, Yingqian Liu, Chuanrui Xu

**Affiliations:** ^1^ School of Pharmacy, Tongji Medical College, Huazhong University of Science and Technology, Wuhan 430030, P. R. China; ^2^ Max Planck Institute for Polymer Research, 55128 Mainz, Germany; ^3^ School of Pharmacy, Lanzhou University, Lanzhou, 730000, P. R. China

**Keywords:** camptothecin, nanocrystal, WCN-21, bottom-up approach, cancer therapy

## Abstract

WCN-21 is a new camptothecin derivative we synthesized and has desirable anti-tumor efficacy, but its aqueous solubility is very low and hurdles the further evaluation and development. In this study, we prepared nanocrystals of WCN-21 through a bottom-up approach to enhance its solubility and obtained WCN-21 nanorods (WND) and nanospheres (WNP). We investigated the crystallization of WND and WNP in different temperature and solvents and found that both temperature and solvents affect the crystal shapes and sizes. We prepared WND at 50°C and DMSO : H_2_O 1: 50 and WNP at 25°C and DMSO : H_2_O 1: 100 and found they were dispersed evenly in water with average hydrodynamic diameters 337 and 231 nm, respectively. WND and WNP increased the solubility of WCN-21 from extreme insolubility to more than 9 and 11 mM in H_2_O or PBS, respectively. *In vitro* studies showed that WND and WNP enhanced the uptake of WCN-21 in tumor cells by 3 and 9 folds, and increased cytotoxicity of WCN-21 in comparison with free WCN-21 by 5 and 6 folds, respectively. In xenograft tumor mice, intravenous injection of WND and WNP enhanced the accumulation of WCN-21 in tumor tissues and improved the anti-tumor efficacy. In addition, WND and WNP did not increase the toxicity of WCN-21 in mice. Therefore, nanocrystal is a robust tool to improve the solubility of insoluble drugs and holds a great potential in the application of drug development.

## INTRODUCTION

Since isolated from Chinese tree *Camptotheca acuminate* in 1960s, 20(S)-Camptothecin (CPT) and its derivatives have been extensively explored as anti-tumor agents [1, 2]. Some of these derivatives, including Irinotecan, SN-38 and Topotecan, have been approved as chemotherapeutic drugs and widely used to treat various human tumors in clinical [3, 4]. However, clinical applications of these CPT derivatives are severely restricted by their heavy toxicity and low stability in plasma [5–7]. Hence, finding novel CPT derivatives with high stability and efficacy but low toxicity is still an unmet clinical need.

Recently, we synthesized a series of new CPT derivatives and found that WCN-21, one of those derivatives, has comparable efficacy but lower toxicity in mice compared with Topotecan (data submitted elsewhere). WCN-21 was synthesized by introducing a thiocarbamide group to the 20′ position of CPT to increase the stability and anti-tumor activity of CPT. Anti-tumor efficacy of WCN-21 was 2 folds higher than Topotecan *in vitro* and was comparable with Topotecan *in vivo*. Moreover, its toxicity was significantly lower than Topotecan in mice (data submitted elsewhere). Thus it is worthy to further explore its therapeutic effects and safety in more animal models. However, like many other CPT derivatives, WCN-21 is water-insoluble, and this insolubility leads to difficulties in administration to animals and restricts the further evaluation and successive development. Therefore, it is required to enhance the solubility of WCN-21 through either chemical modifications or pharmaceutical formulations.

In comparison with the chemical modification, the pharmaceutical approach has several advantages to improve solubility of WCN-21. First, the chemical modification might alter the toxicity of WCN-21 since it requires the introduction of a hydrophilic group to the molecule. Second, chemical modification may affect the stability of lactone ring in WCN-21, which is critical for the anti-tumor activity of camptothecin derivatives [8, 9]. Therefore, further chemical modifications may lead to the loss of anti-tumor activity and increased toxicity of WCN-21. Pharmaceutical improvement, however, will not alter the molecular structure and hence does not affect the inherent activity and toxicity of WCN-21. Hence, pharmaceutical approaches are more appropriate than chemical modifications to improve the solubility of WCN-21.

To improve solubility of drugs, many nano systems have been fabricated and applied, such as liposomes, micelles, silica nanoparticles and dendrimers [10–12]. In our previous work, we also used multiple nano systems to improve the solubility of drugs and found that these nano systems indeed improve the solubility and hence enhance the efficacy [13, 14]. However, the auxiliary ingredients used in these nano systems have potential systemic toxicity and may obstruct the further evaluation of safety of WCN-21 [15].

Nanocrystals, in contrast, contain no auxiliary material and serve as an ideal formulation for WCN-21. Since the nanocrystals offer a drug loading as high as 100% without any encapsulating/solubilizing excipients, [16, 17] it can avoid toxic side effects inflicted by the excipients and also reach therapeutic concentrations at low dose [18]. Moreover, nanocrystals have retention and permeability (EPR) effect as the other nano systems do [19, 20]. Hence, nanocrystals may increase the solubility of WCN-21 and do not affect the evaluation to its druggability.

At present, nanocrystals are produced by either top-down or bottom-up approaches [21, 22]. A top-down approach generally leads to instability of chemicals and transition of crystal forms during the grinding processing. [23] In addition, top-down approaches are time consuming and energy intensive [24, 25]. In contrast, bottom-up approaches were used to grow nanocrystals by injecting a compound solution into antisolvent [15, 26, 27]. This crystal growing process is mild and generally does not affect the structure stability of molecules, which is critically fit for WCN-21 [28, 29].

In this study, we prepared WCN-21 nanocrystals using a bottom-up approach and obtained two forms of nanocrystals, WCN-21 nanorods (WND) and nanospheres (WNP). We compared their uptake, cytotoxicity, pharmaceutical properties and anti-tumor effects with free WCN-21 *in vitro* and *in vivo*. Our study illustrates that the nanocrystal formulation enhances the solubility and anti-tumor effects of WCN-21 without increasing its toxicity and is a feasible approach to improve the solubility of insoluble drugs.

## RESULTS

### Preparation and characterization of WND and WNP

**Figure 1:**
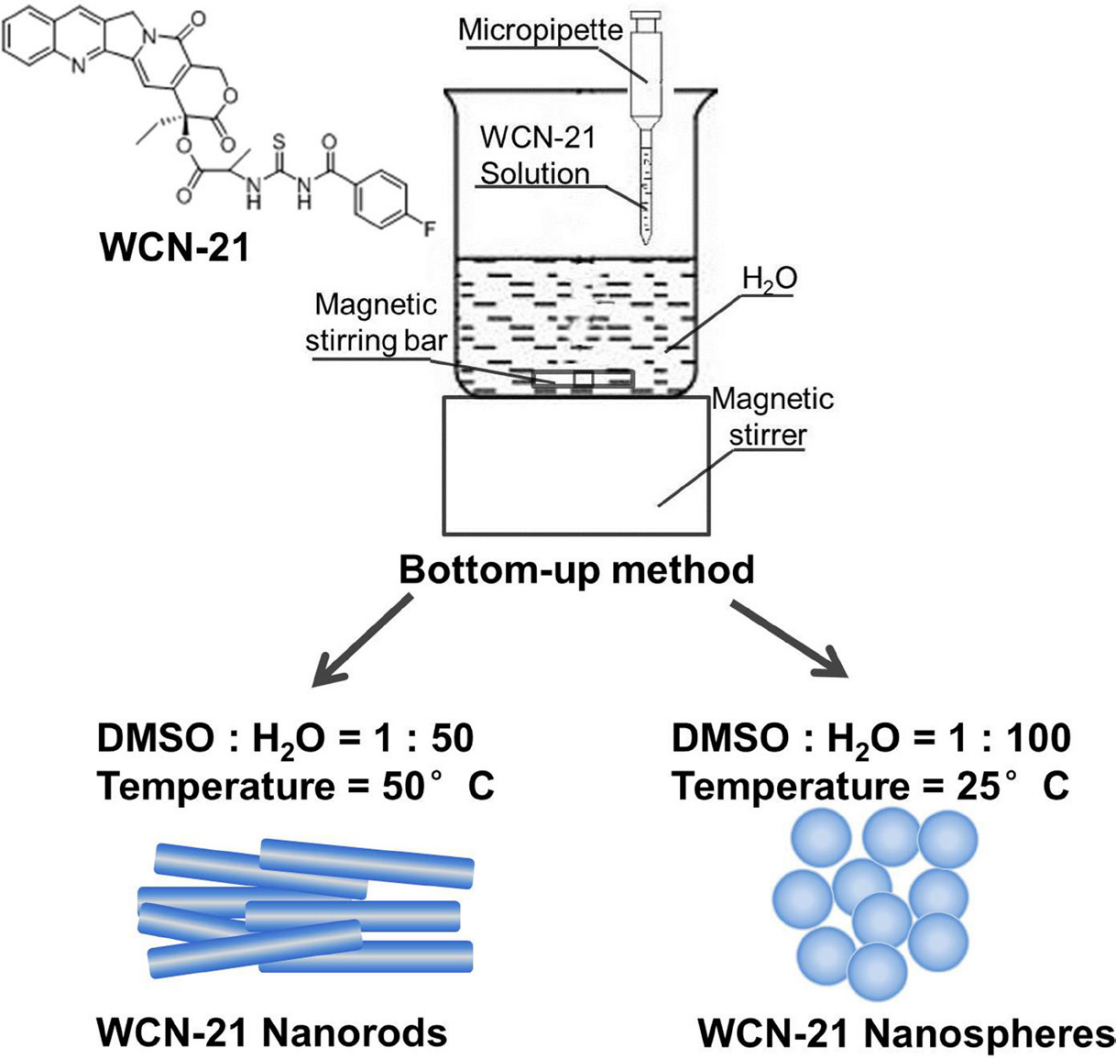
Molecular structure of WCN-21 and illustration of the WND and WNP

**Figure 2:**
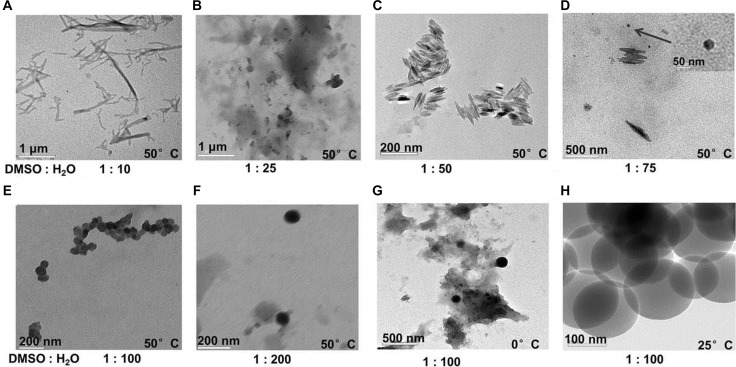
TEM images of WCN-21 nanoparticles or floc formed under different temperature and solvents WCN-21 dissolved in DMSO at 2 mM was added in to H_2_O at various ratios.

To prepare the nanocrystals of WCN-21, we first explored the impacts of ratio of solvents and temperature on the crystallization Figure 1. When the ratio DMSO/H_2_O was at 1 : 10 or 1 : 25, large floc was observed (Figure 2A and 2B). When the ratio DMSO/H_2_O was at 1 : 50 or 1 : 100, WCN-21 nanorods (Figure 2C) and nanospheres (Figure 2E, 2G and 2H) were obtained, respectively. At a DMSO/H_2_O ratio of 1 : 75, both nanorods and nanospheres were observed (Figure 2D). At a DMSO/ H_2_O ratio of 1 : 200, WCN-21 formed nanospheres (Figure 2F).

Temperature also affected the crystallization. At 50°C and a DMSO/H_2_O ratio 1 : 100, inhomogenous WCN-21 nanospheres were obtained (Figure 2E). At 25°C and same DMSO/H_2_O ratio, homogenous WCN-21 nanospheres were obtained (Figure 2H). At 0°C, excessive crystallization resulted in reduced crystal formation (Figure 2G).

**Figure 3:**
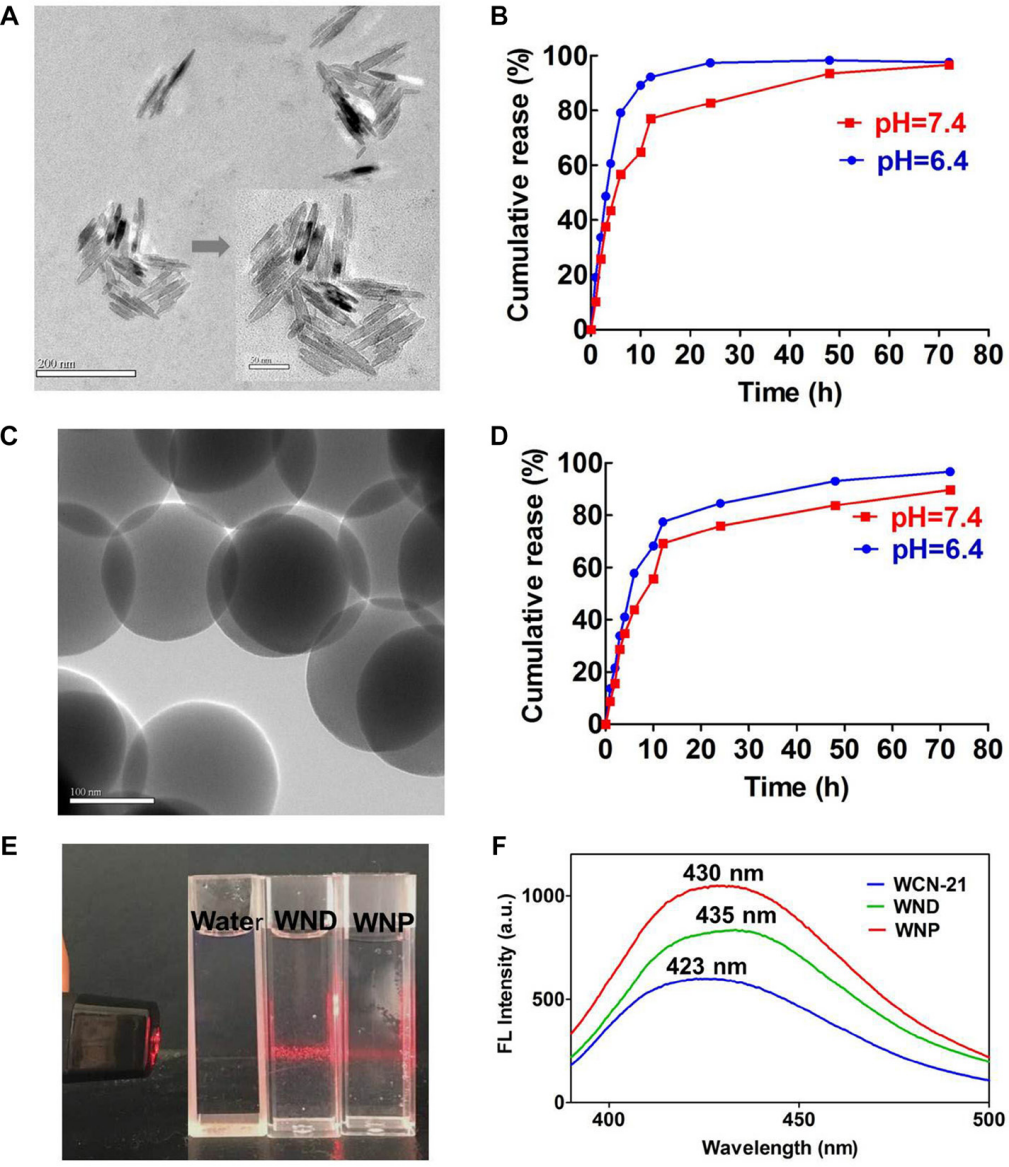
Characterization of the WND and WNP (**A**) TEM image of WND. The larger bar indicates 200 nm and the shorter one indicates 50 nm. (**B**) *In vitro* release of WCN-21 from WND at pH 6.4 and 7.4. (**C**) TEM image of WNP. The bar indicates 100 nm. (**D**) *In vitro* release of WCN-21 from WNP at pH 6.4 and 7.4. (**E**) Tyndall effect of WND (10.2 μM WCN-21) and WNP (4.9 μM WCN-21) in water. (**F**) Emission spectra of WCN-21 (0.1 μM), WND (0.15 μM WCN-21) and WNP (0.2 μM WCN-21). WCN-21 λex_em = 360_ 423 nm, WND λex_em = 360_435 nm, WNP λex_em = 360_430 nm.

According to these results, we then prepared a batch of WCN-21 nanorods (DMSO/H_2_O at 1 : 50, temperature at 50°C) and nanospheres (DMSO/H_2_O at 1 : 100, temperature at 25°C) and characterized those nanocrystals. The average particle diameters of WND and WNP were then measured using transmission electron microscope (TEM) and analyzed using software Nano Measurer 1.2.0 (http://nano-measurer.software.informer.com/). The results showed that average particle diameters of WND and WNP were 162.70 ± 4.3 and 106.25 ± 2.9 nm, respectively (Table 1). Dynamic light scattering (DLS) analysis showed that the average hydrodynamic diameters of WND and WNP were 336.9 ± 17.9 nm and 230.6 ± 7.8 nm, respectively (Table 1 and Supplementary Figure 1). The polydispersity indexes (PDI) of WND and WNP were 0.304 and 0.258, respectively, indicating narrow size distributions of those particles. Zeta potential of WND and WNP were −3.99 ± 1.04 and −8.57 ± 3.13 mV, respectively (Table 1 and Supplementary Figure 1). TEM images also demonstrated that WND and WNP were dispersed evenly in the solution, with uniform particle shapes (Figure 3A and 3C). Critically, WCN-21 is extremely insoluble in pure water and 5 mM in H_2_O with 5% DMSO as co-solvent. In contrast, WND and WNP significantly increased its solubility to more than 9 mM and 11 mM in H_2_O or PBS without any co-solvent. In addition, we did not observe significant changes of size, zeta potential and PDI after 7 days storage of WNP or WND in H_2_O (Supplementary Figure 2).

**Table 1:** Key parameters of WND and WNP

Nanocrystals	Inorganic size (Nano Measurement)/nm	Hydrodynamic size (DLS)/nm	PDI	Zeta potential (mV)
WND	162.70 ± 4.3 (long)	336.9 ± 17.9	0.304	−3.99 ± 1.04
	13.57 ± 2.1 (thick)			
WNP	106.25 ± 2.9	230.6 ± 7.8	0.258	−8.57 ± 3.13

Data are expressed as mean ± SD of three independent samples.

Considering that the micro-environment in tumor tissues tends to be more acidic (pH 5.0–6.5) than that in normal tissues (pH 7.4), we examined the release properties of WCN-21 from WND or WNP in PBS with low pH values. As exhibited in Figure 3B and 3D, release of WCN-21 from WND or WNP at pH 6.4 was more rapid and efficient than that at pH 7.4, suggesting acid liable release properties of WND and WNP. In addition, aqueous solution of WND and WNP showed typical Tyndall light scattering (Figure 3E). We examined the fluorescence spectrum of WCN-21 in a H_2_O/DMSO mixture using a fluorescence spectrophotometer and found a red-shift of the maximum emission from 423 (WCN-21) to 435 (WND) and 430 nm (WNP), respectively (Figure 3F). These results indicate that WCN-21 was self-assembled to nanocrystals as expected.

### Uptake and anti-tumor activity of WND and WNP *in vitro*

**Figure 4:**
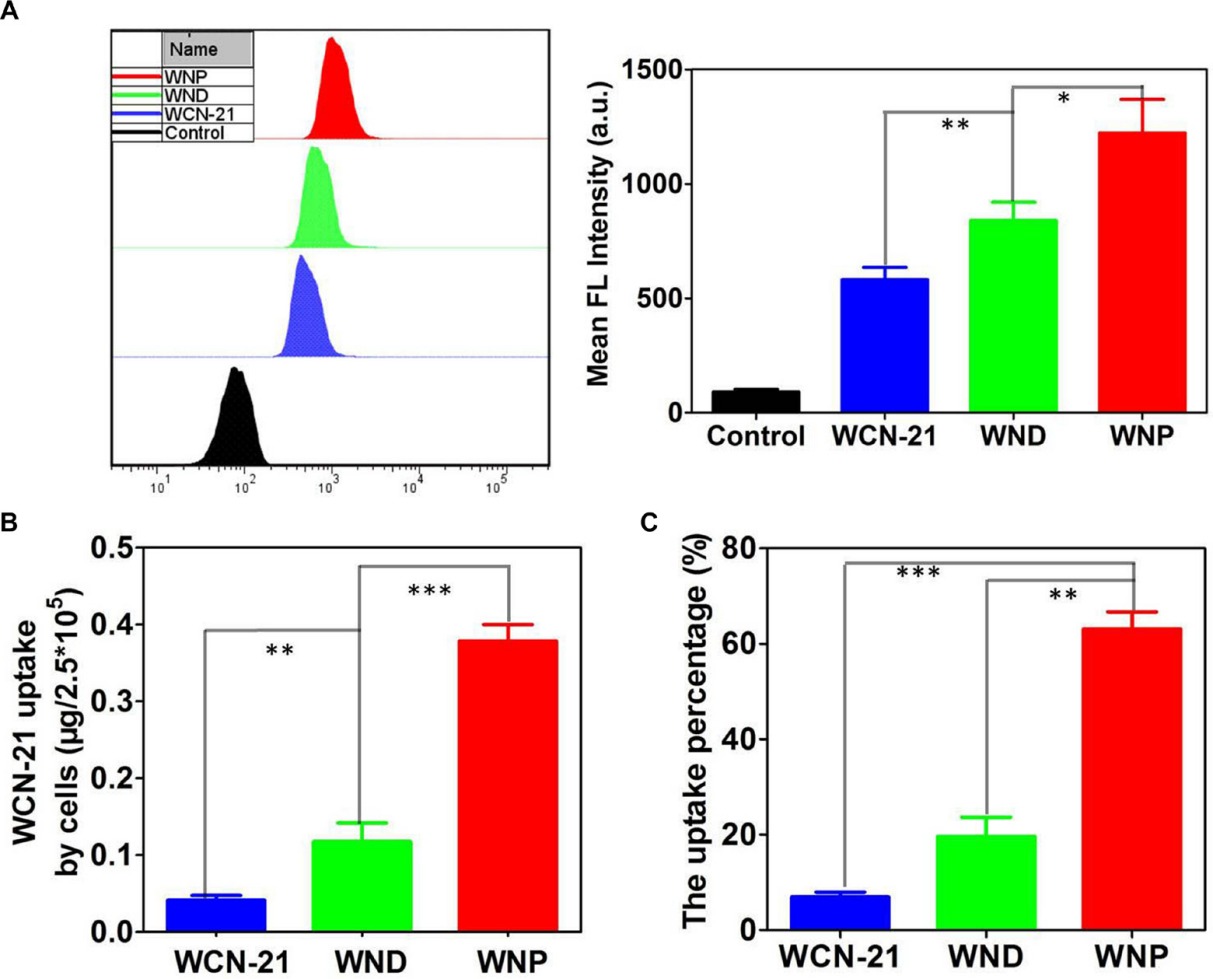
Intracellular uptake and accumulation of WCN-21 (**A**) Uptake of WND or WNP in HepG2 cells determined by flow cytometry. HepG2 cells were incubated with WCN-21(10 μM), WND (10 μM WCN-21) or WNP (10 μM WCN-21) for 4 h and then collected for flow cytometry analysis. Left panel shows the uptake of WCN-21, WND or WNP in treated HepG2 cells. Right panel is the quantification of mean fluorescence intensity of HepG2 cells using flow cytometry analysis. (**B**) Fluorescence spectrophotometer analysis of the amount of WCN-21 in 2.5 × 10^5^ HepG2 cells incubated with WCN-21 (0.5 μM), WND (0.5 μM WCN-21) or WNP (0.5 μM WCN-21) for 4 h. (**C**) The percentage of WCN-21 taken up by HepG2 cells. The uptake percentage of WCN-21 was determined by the mass of WCN-21 in the cells versus the initially given amounts of WCN-21. The experiments are repeated 3 times and data are presented as average ± standard error. The statistical significance level is *p < 0.05, **p < 0.01 and ***p < 0.001.

To determine the uptake of WND and WNP in tumor cells, we treated HepG2 cells with WCN-21, WND or WNP at a WCN-21 concentration of 10 μM. Flow cytometry analysis showed that both WND and WNP promoted the uptake of WCN-21 in HepG2 cells compared with free WCN-21 (Figure 4A). Fluorescence spectrophotometer examination revealed that WCN-21 contents were 0.073, 0.151 and 0.441 μg/2.5 × 10^5^ cells in WCN-21, WND or WNP treated HepG2 cells, respectively (Figure 4B). The uptake percentage of WCN-21 was determined by the ratio of WCN-21 amounts in the cells to the initially given amounts of WCN-21. The result showed that uptake percentages of WND and WNP were 19.6% and 63.0%, 3 and 9 folds of free WCN-21 (6.9%), respectively (Figure 4C). These data suggest that WND and WNP can increase the uptake and accumulation of WCN-21 in tumor cells compared with free WCN-21.

**Figure 5:**
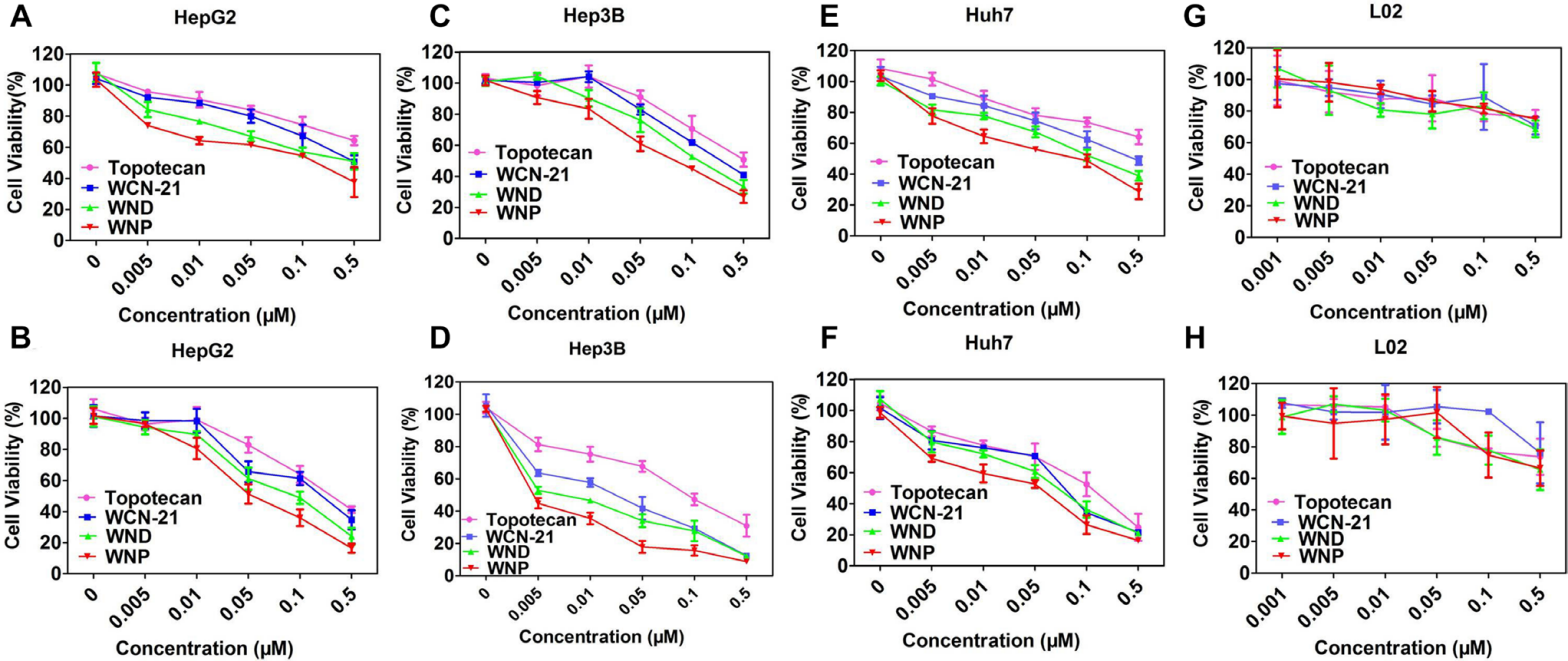
Cytotoxicity of Topotecan, WCN-21, WND and WNP in HepG2 (**A**, **B**), Hep3B (**C**, **D**), Huh7 cells (**E**, **F**) and L02 (**G**, **H**). The survival of cells was determined by MTT assay and the data are presented as average ± standard error (*n* = 3). Panel A, C, E and G show the results of 24 h treatment and panel B, D, F and H show the results of 48 h treatment, respectively.

We detected the cytotoxicities of WND and WNP in HepG2, Hep3B, Huh7 or L02 cells. We treated those cells with drugs for 24 or 48 h and determined the viability using MTT assay. In all tumor cell lines, WCN-21 showed comparable cytotoxic activity with free Topotecan. However, WND and WNP have much lower IC_50_ than free WCN-21 in all tumor cells, suggesting increased cytotoxicities of WND and WNP (Figure 5 and Table 2). Furthermore, prolonging the treatment time from 24 to 48 h improved the cytotoxic effect of WND and WNP, suggesting that WND and WNP maintained a sustaining release of WCN-21 in tumor cells (Figure 5 and Table 2). Of note, the IC_50_ values of Topotecan, WCN-21, WND and WNP were higher than 0.5 mM in L02 cells (Figure 5G, 5H and Table 2). These results suggest that cytotoxicity of WCN-21, WND or WNP on normal cell lines is low.

**Table 2:** IC_50_ of WCN-21, WND and WNP in different cell lines with different treatment time

Cell lines		Topotecan	WCN-21	WND	WNP
Hep3B	24 h	0.4616 ± 0.02	0.27 ± 0.010	0.1674 ± 0.0032	0.09328 ± 0.0013
	48 h	0.1121 ± 0.0024	0.01838 ± 0.0028	0.007459 ± 0.001	0.003129 ± 0.0017
HepG2	24 h	1.594 ± 0.0206	0.5014 ± 0.0036	0.4035 ± 0.037	0.1375 ± 0.0062
	48 h	0.2847 ± 0.009	0.1836 ± 0.049	0.1007 ± 0.0038	0.05663 ± 0.058
Huh 7	24 h	0.5 ± 0.069	0.4017 ± 0.009	0.1719 ± 0.0032	0.07261 ± 0.0002
	48 h	0.1167 ± 0.048	0.07221 ± 0.012	0.05807 ± 0.0201	0.02625 ± 0.0405
L02	24 h	> 0.5	> 0.5	> 0.5	> 0.5
	48 h	> 0.5	> 0.5	> 0.5	0.490 ± 0.093

Data are expressed as mean ± SD of three independent samples.

### WND and WNP induce apoptosis and cell cycle arrest in HepG2 cells

**Figure 6:**
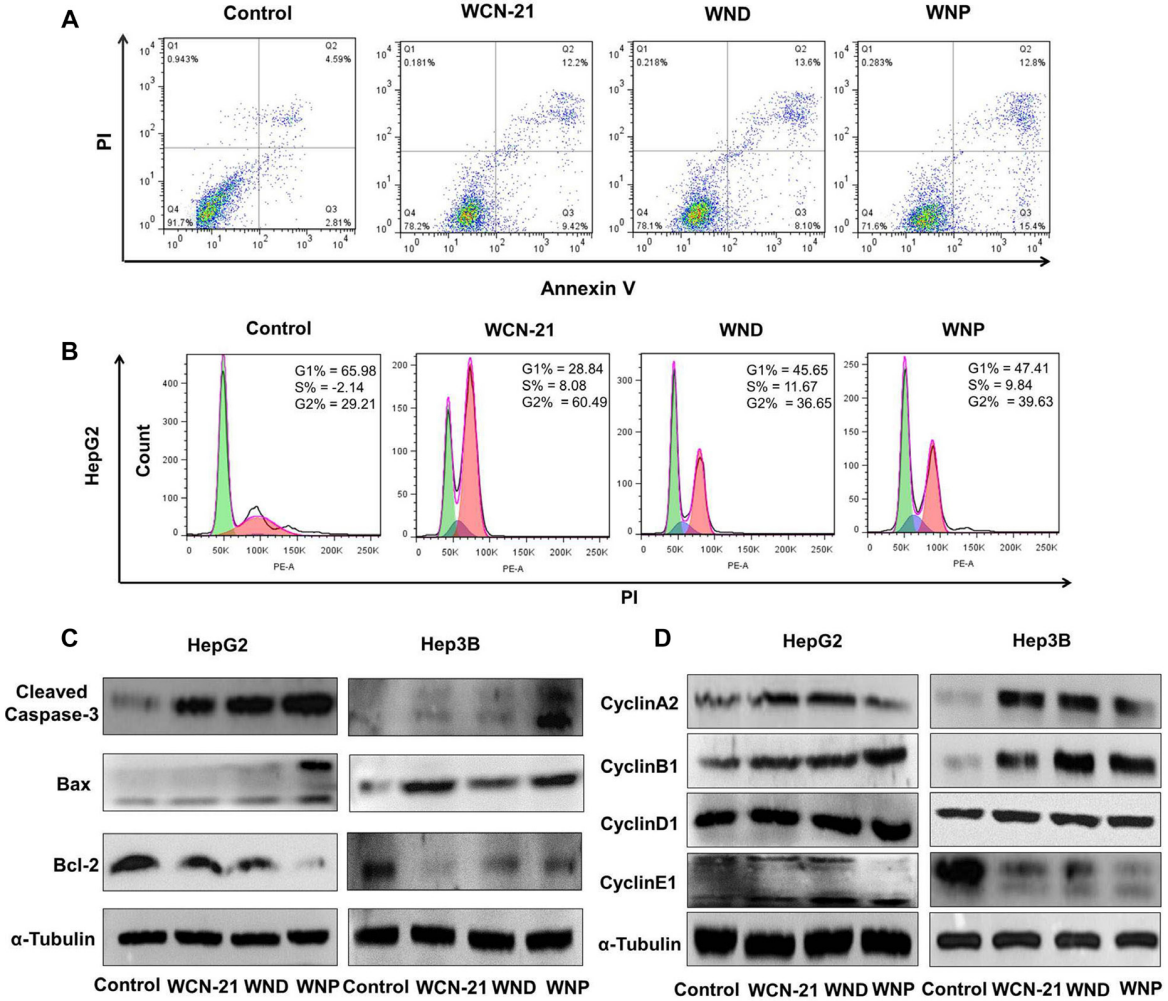
Effects of WCN-21, WND and WNP on tumor cell apoptosis and cell cycle progression *in vitro*. HepG2 or Hep3B cells were treated with WCN-21, WND or WNP at a concentration of 0.02 μM WCN-21 for 24 h and then harvested for flow cytometry or western blotting analysis (**A**) Apoptosis of HepG2 cells induced by WCN-21, WND or WNP. Inserted numbers in the profiles indicate the percentage of the cells present in this area. Lower left, living cells; lower right, early apoptotic cells; upper right, late apoptotic cells; upper left, necrotic cells. (**B**) Distribution histograms of HepG2 cells in various cell cycle phases. (**C**) The expression levels of apoptosis-related proteins in HepG2 or Hep3B cells treated with WCN-21, WND or WNP. (**D**) The expression levels of cell cycle-related proteins in HepG2 or Hep3B cells induced by WCN-21, WND and WNP. Untreated cells were used as a blank control, and α-Tubulin was used as a loading control.

To explore whether WND and WNP exert anti-tumor effect through the mechanism as WCN-21, we treated HepG2 cells with low dose WCN-21, and equivalent dose WND or WNP. After 24 h treatment, 0.02 μM WCN-21 induced apoptosis in 19.08% HepG2 cells, whereas the vehicle induced only 7.4% apoptosis in treated cells (Figure 6A). WND treatment induced apoptosis in 21.7% HepG2 cells, while WNP induced apoptosis in 28.2% HepG2 cells (Figure 6A). This result indicates that WND and WNP promote the apoptosis of HepG2 cells in a marginal strength in comparison with WCN-21. We then examined the effects of WND and WNP on cell cycle progression of HepG2 cells. Cell cycle analysis by FACS showed that HepG2 cells treated with WCN-21, WND or WNP were all arrested in G2 phase, suggesting that anti-tumor activity of WND or WNP was dependent on release of WCN-21 (Figure 6B). To confirm those phenotypic observations in molecular level, we detected expression of apoptosis and proliferation related genes. In both HepG2 and Hep3B cells, WND and WNP treatment significantly increased expression of Bax and cleaved Caspase-3 as WCN-21 did (Figure 6C). Correspondingly, anti-apoptosis gene Bcl-2 was down-regulated upon WCN-21, WND or WNP treatment in both HepG2 and Hep3B cells (Figure 6C). Likewise, G2 phase related cyclin A2 and cyclin B1 were accumulated upon WCN-21, WND or WNP treatment due to cell cycle arrest in G2 phase (Figure 6D). Correspondingly, G1 phase related cyclin E1 was decreased in treated cells (Figure 6D). Taken together, these results indicate that though WND and WNP enhanced anti-tumor effects of WCN-21, their anti-tumor mechanism was consistent with that of WCN-21.

### Distribution, pharmacokinetics and anti-tumor effects of WCN-21, WND and WNP in mice

**Figure 7:**
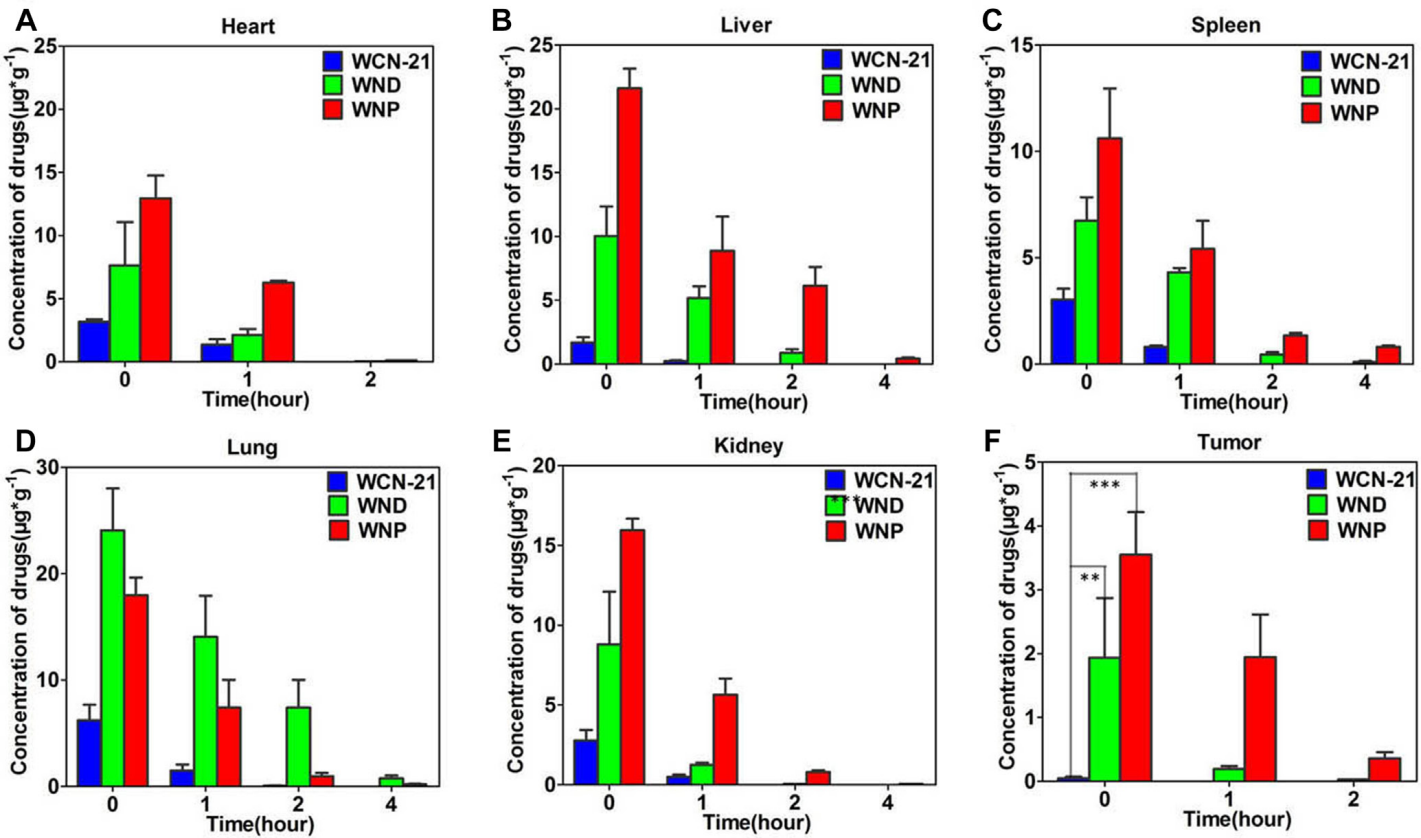
Distribution of WCN-21, WND, and WNP in xenograft mouse model Mice were injected (i.v.) with WCN-21 (4 mg/kg), WND (4 mg/kg WCN-21), or WNP (4 mg/kg WCN-21) and tissues were collected at 0, 1, 2, and 4 h post injection. Organs (**A** to **E**) or tumor (**F**) samples were homogenized and drugs were extracted for content analysis of WCN-21 using a fluorescence spectrometer. Data are presented as average ± standard error (*n* = 3), and the statistical significance level is **p < 0.01 and ***p < 0.001.

To compare the distribution of WND and WNP with WCN-21 in mouse tissues, we injected (i.v.) tumor bearing mice with WCN-21, WND or WNP and then examined their distribution. WCN-21, WND or WNP was injected to xenograft tumor mice at a dose of 4 mg/kg WCN-21 and then mouse tissues were collected and analyzed after sacrifice of mice. Compared with free WCN-21, WND and WNP injections significantly increased the amount of WCN-21 in mouse hearts, livers, spleens, lungs and kidneys by 2–3 folds and 3–10 folds, respectively (Figure 7 and Supplementary Figure 3). In addition, the amount of free WCN-21 in tumor tissue was less than 0.1 μg/g tissue (0.09% ID/g), significantly lower than that of WND (1.94 μg/g, 2.4% ID/g) and WNP (3.55 μg/g, 4.4% ID/g) at 0 min after injections. At 1 and 2 h post injections, WNP demonstrated a better retention in tumor tissues compared with WND, with average concentrations of 1.95 and 0.19 μg/g tissue, respectively. These results indicate that both WND and WNP can increase the accumulation of WCN-21 in tumor tissues through EPR effects.

**Figure 8:**
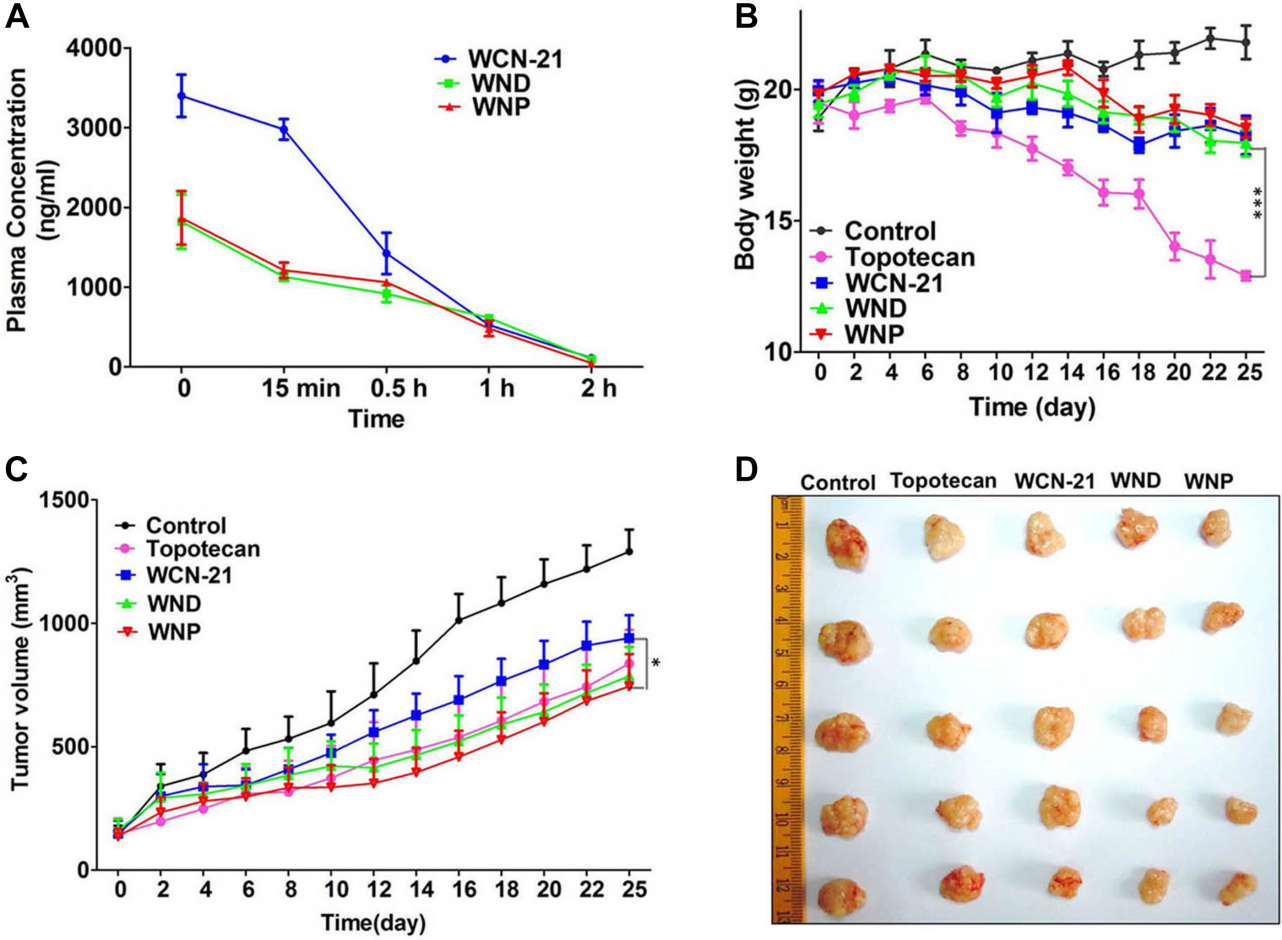
The pharmacokinetic analysis of WCN-21 in mouse plasma in Kunming mice and anti-tumor effects of WCN-21, WND and WNP in xenograft mouse model (**A**) Pharmacokinetic curves of WCN-21, WND and WNP in the mouse plasma. The female Kunming mice were injected intravenously by a single dose of drugs at a WCN-21 concentration of 4 mg/kg and the blood samples (*n* = 3) were collected at 5 time points. (**B**) Body weight changes of mice treated with free WCN-21, WND or WNP by 5 doses tail vein injection (4 mg/kg WCN-21). Saline injection was used as the control. Data are expressed as mean ± SD (*n* = 5). (**C**) Growth curves of xenograft tumors treated with saline, free WCN-21, WND or WNP by a single dose tail vein injection (4 mg/kg WCN-21). The curves present the changes of tumor sizes from the day of injection (day 0). Data are expressed as mean ± SD (*n* = 5). *p < 0.05. (**D**) Excised tumors at the end point of the experiment (day 25 after the drug injection).

Next, we examined the pharmacokinetics of WND and WNP and obtained their plasma clearance kinetics. The C_max_ of WND (1820 ng/ml) and WNP (1868 ng/ml) were much lower than that of free WCN-21 (3398 ng/ml). The t_1/2_ of WND and WNP was 2 folds of that of WCN-21 (Figure 8A).

We then evaluated anti-tumor efficacy of WCN-21, WND and WNP using a HepG2 xenograft tumor mouse model. WCN-21, WND or WNP was administrated by tail vein injection at the concentration of 4 mg/kg WCN-21 when the subcutaneous tumors grew to ~50 mm^3^. Tumor volume and mouse body weights were recorded during drug administration period. The mice were sacrificed at 25th day after the injection and tumors were collected for histology examination. At 25th day, tumor sizes of WCN-21 group mice (939 mm^3^) were relatively higher than that of Topotecan group mice (837 mm^3^). However, tumors in WND group (786 mm^3^) and WNP group (745 mm^3^) were smaller than that in Topotecan group (Figure 8C). The tumor morphology also showed that WND and WNP have more robust anti-tumor efficacy compared with free WCN-21 (Figure 8D). Hence, these results indicate that WND and WNP enhanced the anti-tumor effect of WCN-21.

### WND and WNP inhibit tumor growth by inducing apoptosis

**Figure 9:**
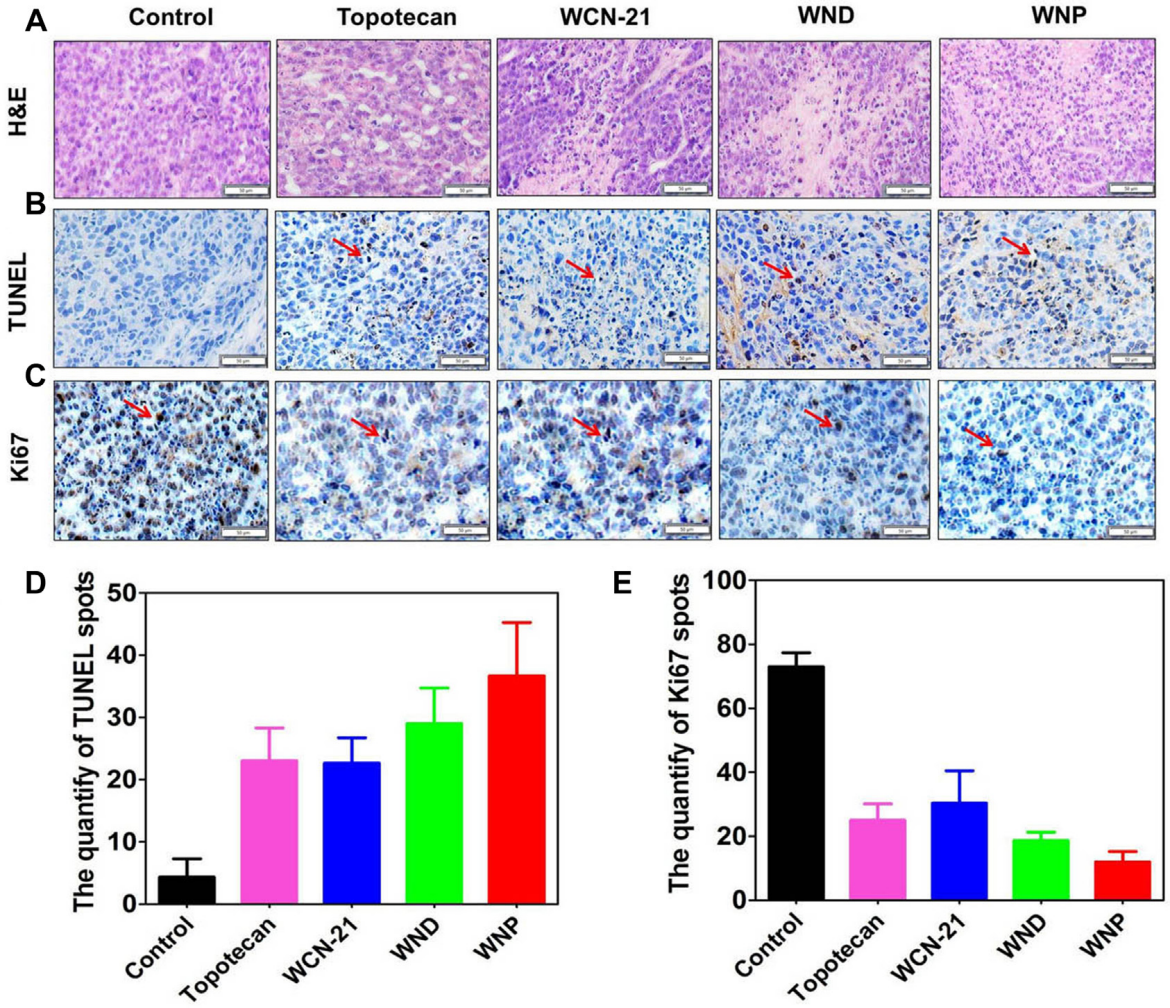
Immunohistochemical analysis of tumor tissues treated with 5 doses injection of Topotecan (4 mg/kg), WCN-21 (4 mg/kg), WND (4 mg/kg WCN-21), or WNP (4 mg/kg WCN-21). The tumors were resected from the mice used for efficacy study and biopsies were made after 4% PFA fixation. The bar indicates 50 μm (**A**) Tumors were sectioned and stained with H&E. (**B**) TUNEL staining of tumor tissues to assess tumor apoptosis. The red arrows show the TUNEL signal. (**C**) Ki67 staining of tumor tissues to assess tumor proliferation. The red arrows indicate Ki67 signal. (**D**) The quantification of TUNEL staining spots. (**E**) The quantification of Ki67 staining. For each treatment, 3 independent fields from different biopsies were counted.

To further assess the anti-tumor mechanism of WND and WNP, we performed histological analysis in tumor tissues treated with WND or WNP. Compared with saline, all of WCN-21, WND, WNP and Topotecan led to coagulation necrosis, cellular crenulation and nucleus vanish in tumor tissues (Figure 9A). Considering these pathological features are typical traits of apoptosis, we examined the apoptotic activity in tumor samples using TUNEL assay. We found that no TUNEL staining was observed in saline injected mice, while robust TUNEL signaling was widespread in all the drugs treated tumors (Figure 9B). In addition, WND and WNP induced more apoptosis than free WCN-21 in tumors, which is consistent with the efficacy study (Figure 9D). Furthermore, Ki67 staining also indicated that tumor cell proliferation in WCN-21 treated mice was comparable with that in Topotecan treated mice, but it was faster than that in WND and WNP treated mice (Figure 9C, 9E). These data collectively suggest that WND and WNP inhibit the tumor growth by inducing apoptosis, which is identical with the mechanism of WCN-21.

### Toxicity of WND and WNP in mice

**Figure 10:**
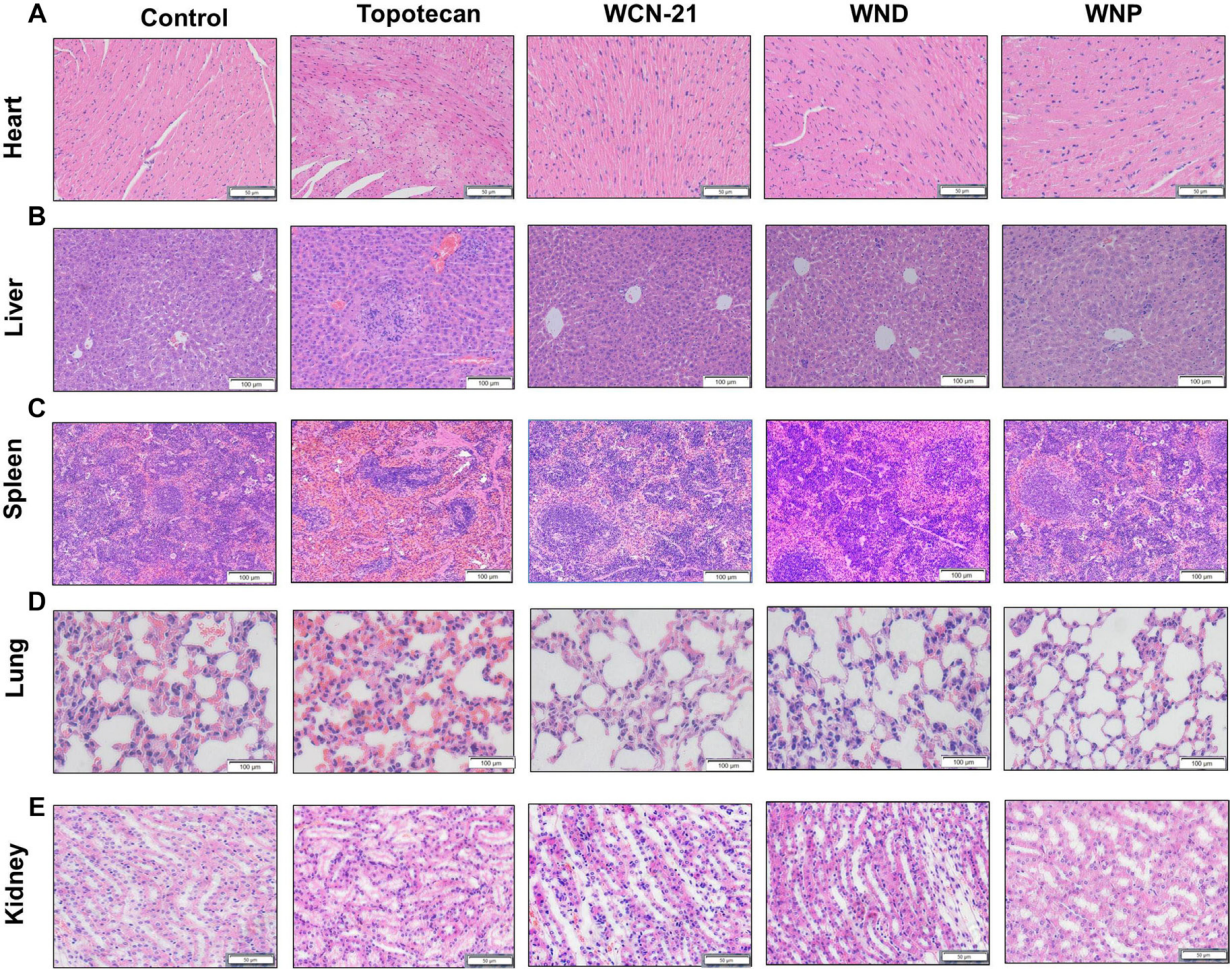
Histological analysis of hearts, livers, spleens, lungs and kidneys from xenograft mouse model treated with saline, Topotecan (4 mg/kg), WCN-21 (4 mg/kg), WND (4 mg/kg WCN-21) or WNP (4 mg/kg WCN-21) The organs were resected from the mice for efficacy study after sacrifice and tissues were collected for histological analysis. The bars indicate 50 μm in heart and kidney panels, 100 μm in liver, spleen and lung panels.

To determine the toxicity of WND and WNP, we collected tissues from the mice used in efficacy study for histological analysis. Compared with control mice, Topotecan treated mice exhibited myocardial fiber fracture and cardiocytes necrosis in hearts, inflammatory infiltration in livers, atrophied white pulp in spleens and disorganization of renal corpuscles in kidneys. Compared with Topotecan, WCN-21 did not produce significant injury in those organs (Figure 10). As expected, WND and WNP also did not show any toxicity in mice (Figure 10). Moreover, all mice in Topotecan group had a significant body weight loss, while mice in WCN-21, WND or WNP group had no apparent body weight loss (Figure 8B). These results indicate that Topotecan had certain toxicity while WCN-21, WND or WNP had little or no toxicity in mice during the experimental period.

## DISCUSSION

Solubility is a crucial parameter for a chemical in term of druggability. Many camptothecin derivatives are hampered to be transformed into clinical studies due to low solubility. In previous studies, we synthesized a new camptothecin WCN-21, which has higher anti-tumor activity than Topotecan. However, its low solubility restricted the further evaluation. In this study, we prepared nanocrystals of WCN-21 and found that those nanocrystals could improve the solubility of WCN-21 significantly. Our study confirmed the feasibility using nanocrystals to enhance the solubility of insoluble drug.

During the preparation, we found that the yield and shape of WCN-21 nanocrystals were affected by both the ratio DMSO/H_2_O and temperature. A possible explanation was that those two factors affected the crystallization of WCN-21. There are two crucial steps during crystallization: nucleation and crystal growth rate [30]. Both the nucleation and crystal growth depend on the level of supersaturation [31]. Compared with the crystal growth rate, the nucleation rate is more dependent on supersaturation [32]. Increasing the supersaturation level leads to augmented nuclei formation and subsequently decreased crystal size [33].

Our result indicates that ratio of solvents affected the supersaturation and subsequently the particle sizes. DMSO/H_2_O at 1:10 or 1:25 was good solvent for WCN-21 and led to low degree of supersaturation. Therefore, WCN-21 molecules formed large floc rather than nanocrystals because of fewer nuclei being formed (Figure 2A and 2B). In contrast, DMSO/H_2_O ratio at 1 : 50 or 1 : 100 increased supersaturation of WCN-21, and led to formation of nanorods (Figure 2C) or nanospheres (Figure 2E, 2G and 2H), respectively. However, further increasing supersaturation (DMSO/H_2_O 1 : 200) did not show impact on the crystallization, possibly because nucleation rate approached maximum value (Figure 2F). Of note, both nanorods and nanospheres were observed at a DMSO/H_2_O ratio of 1 : 75, suggesting that crystal shapes were also associated with supersaturation (Figure 2D). Taken together, we conclude that reducing the proportion of good solvent in a specific range leads to increased supersaturation and then enhances crystal formation. In addition, degree of supersaturation also affects the crystal shapes.

Our results also demonstrated that temperature had influence on the WCN-21 supersaturation level. Decreasing the temperature led to increased supersaturation (Figure 2). At DMSO/H_2_O ratio 1:100 and 50°C, the solubility of WCN-21 in the DMSO/H_2_O is higher than that at 25°C and resulted in a lower degree of supersaturation. Furthermore, higher temperature led to lower crystal growth rate and inhomogenous WCN-21 nanospheres (Figure 2E). At 25°C, supersaturation increased, and homogenous WCN-21 nanospheres were obtained (Figure 2H). However, too low temperature did not favor the formation of WCN-21 nanocrystals. At 0°C, too rapid crystallization resulted in decreased formation of regular nanocrystals (Figure 2G). Taken together, optimization of the antisolvent/solvent ratio and temperature were critical for the preparation of WCN-21 nanocrystals and this finding may be applicable to other camptothecin derivatives.

Compared with free WCN-21, both WND and WNP showed a higher uptake in tumor cells and xenograft tumor tissues. Analyses using flow cytometry and fluorescence spectrophotometer showed that the amount of WND and WNP were significantly higher than that of free WCN-21 in HepG2 cells (Figure 4). It suggests that WCN-21 nanocrystals indeed improve the uptake and accumulation of WCN-21 in cancer cells.

Of note, the uptake percentage of WNP was higher than that of WND in tumor cells. This phenomenon was potentially due to different size, charge, and shape between the WND and WNP. Since WND and WNP have similar zeta potential, size and shape are two factors affecting their uptake efficiency. It has been reported that in the same hydrodynamic size nanorods penetrate tumors more rapidly than nanospheres [34]. In addition, smaller size is beneficial for nanoparticle to enter cells [35]. Considering WNP (hydrodynamic size 230.6 ± 7.8 nm) was smaller than WND (336.9 ± 17.9 nm), higher uptake efficiency of WNP was potentially due to its smaller size.

The sizes of WND and WNP also affected their tissue distribution and pharmacokinetics. For instance, the distribution of WND in mouse lungs was higher than that in WNP. It was reported that the distribution of nanocrystals in mouse organs following i.v. injection is depending on their particle size and composition [36]. Because the components of WND and WNP were the same, we speculate that the higher distribution of WND in mouse lungs was attributed to its larger size. The distinct pharmacokinetic properties of WND and WNP from WCN-21 were also possibly attributed to their larger sizes (> 200 nm), which led to slow dissolution in plasma [36]. Consequently, nanocrystals are recognized as foreign matters by phagocytic cells of mononuclear phagocyte system (MPS) and cleared rapidly from plasma. This leads to rapid decrease of WCN-21 in plasma after WND or WNP injection in the first minutes. Subsequently, phagocytic cells transferred those nanocrystals to RES (reticuloendothelial system) organs such as liver, spleen and lungs and resulted in high accumulation of WND and WNP in those organs. Similar results was obtained by Gao, L., et al. [37]

Furthermore, both WND and WNP increase the accumulation of WCN-21 in tumor tissues compare with free WCN-21. Efficient uptake of WCN-21 produced better anti-tumor effects both *in vitro* and *in vivo*. WND and WNP have lower IC_50_ *in vitro* and better tumor suppression capability than WCN-21 and Topotecan (Figure 5 and Table 2). *In vivo*, WND and WNP showed significantly higher distribution in tumor tissues than free WCN-21, thus profoundly enhanced the anti-tumor effects of WCN-21. Taken together, these data suggest that the WND and WNP enhance cytotoxic effects of WCN-21 by increasing its uptake in tumor cells and tissues.

Taken together, our study, for the first time, demonstrated that a camptothecin derivative WCN-21 solely self-assembled into two different types of nanocrystals. Both nanocrystals not only improved the solubility of WCN-21 and its distribution in tumor tissues, but also enhanced its anti-tumor efficacy. Therefore, our study demonstrated that nanocrystal technique is effective to improve the solubility and efficacy of new camptothecin derivatives. The preparation and evaluation methods described in this manuscript could be applied for the other insoluble drugs.

## MATERIALS AND METHODS

### Materials

WCN-21 (purity > 95%) was synthesized in our lab. Dimethyl sulfoxide (DMSO) and methyl alcohol were purchased from Sinopharm Chemical Reagent Co., Ltd (Shanghai, China). 3-(4, 5-dimethyl-thiazol-2-yl)-2, 5-diphenyl tetrazolium bromide (MTT) was purchased from Sigma-Aldrich Chemical Co. (St Louis, MO, USA). All solvents and reagents were of analytical or HPLC grade and used without further purification.

Dulbecco’s modified eagle medium (DMEM) was purchased from Sigma-Aldrich (St. Louis, MO, USA). Fetal bovine serum (FBS) was purchased from Zhejiang Tianhang Biological Technology (Hangzhou, China). M-plasmocin was purchased from Invivogene (San Diego, CA, USA). Cell lines HepG2, Hep3B, Huh7, and L02 were obtained from the China Center for Type Culture Collection at Wuhan University (Wuhan, China).

### Cell culture

Tumor cell lines were cultured with high glucose DMEM supplemented with penicillin, streptomycin, and 10% FBS in 37°C and 5% CO_2_ incubators. M-plasmocin at a concentration of 2.5 μg/ml was used to prevent the possible mycoplasma infections.

### Mice

Balb/C nude mice (~20 g, 6~8 weeks old) were obtained from Beijing Huafukang Bioscience Technology (Beijing, China). Female Kunming mice (body weight ~25 g, 6~8 weeks old) were obtained from Laboratory Animal Center of the Huazhong University of Science and Technology. All mice were kept in filter-topped cages with standard rodent chow, water available ad libitum and a 12 h light/dark cycle. The experiment protocol was approved by Committee on Ethical Animal Experiment at Huazhong University of Science and Technology.

### Preparation of WND and WNP

WND and WNP were prepared by a bottom-up method. WCN-21 was dissolved in DMSO to a concentration of 2 mM. Then the solution was sonicated to ensure that WCN-21 was totally dissolved in DMSO. To prepare WND, 5 ml of water was heated to 50°C and then 100 μl of 2 mM WCN-21 solution was rapidly injected into the water with continuous stirring at 50°C for 5 min. To prepare WNP, 5 ml of water was heated to 25°C and then 50 μl of 2 mM WCN-21 solution was rapidly injected into the water with continuous stirring at 25°C for 5 min.

After preparation of WND or WNP, the nanocrystals were centrifuged at 12000 × g for 30 min at room temperature and supernatant containing DMSO was discarded to collect nanocrystals. Then nanocrystals were resuspended in the H_2_O or PBS. Both WND and WNP solution were stored at 4°C and protected from light.

### Characterization of WND and WNP

Particle sizes and zeta potential of WND and WNP were measured by zeta potential analyzer (Zeta PALS, Brookhaven Instruments Corporation, Austin, TX) according to the manufacturer’s instructions. All measurements were carried out at room temperature. Each parameter was measured 3 times; average values and standard deviations were calculated. WND and WNP were observed and photographed using a transmission electron microscope (JEOL 100CX II TEM, Japan).

*In vitro* pH-dependent release of WCN-21 from WND or WNP was studied using a dialysis method at 37°C. Phosphate-citrate buffered saline (PBS-citrate) at pH 7.4 and 6.4 was used as the drug-release media to simulate normal tissue and tumor environment, respectively. Dialysis bags (MW cutoff 1,000 Da) containing WND or WNP were placed into brown bottles containing 8 ml of PBS-citrate at pH 7.4 or 6.4. These bottles were shaken at 37°C while shielded from light. Samples were collected at various intervals and equal volume of fresh buffer was supplied each time. The concentration of released WCN-21 was measured on a Hitachi F-4500 fluorescence spectrophotometer (Tokyo, Japan) and calculated according the standard curve of WCN-21.

### Uptake of WCN-21 measured by flow cytometry

HepG2 cells were seeded in 12-well plates at a density of 5 × 10^4^ cells per well and then incubated with WCN-21 (10 μM), WND (10 μM WCN-21) and WNP (10 μM WCN-21) for 4 h. After harvested, cells were washed with phosphate-buffered saline (PBS), and the fluorescence was measured using a flow cytometer (BD LSRFortessa X-20) with an excitation wavelength of 360 nm. For each test, 2 × 10^4^ cells were counted.

### Uptake of WCN-21 measured by fluorescence spectrophotometer

HepG2 cells were seeded in 6-well plates at a density of 1.5 × 10^4^ cells per well and then incubated with WCN-21 (0.5 μM), WND (0.5 μM WCN-21) and WNP (0.5 μM WCN-21) for 4 h. After harvesting, cells were washed with PBS and then centrifuged at 1000 × g for 3 min at room temperature to collect the cells. After that, cells were resuspended with 100 μl PBS and then sonicated at 100 W for 20 s (intermittent) under the ice bath in three cycles. Then, 100 μl methyl alcohol and 200 μl DMSO were added in and the mixture was vortexed for 1 min. Vortexed solution was centrifuged at 12000 × g for 5 min at room temperature. Finally, supernatant was collected for fluorescence analysis with a fluorescence spectrophotometer (Hitachi F-4500, software FL solution, Japan). A standard curve was determined using a series of dilutions of free WCN-21. The concentration of WCN-21 in cell lysate was calculated using the standard curve of WCN-21.

### MTT Assay

HepG2, Hep3B, Huh7 or L02 cells were seeded in 96-well plates at a density of 5 × 10^3^ cells per well. After 24 h, cells were treated with various concentrations of WCN-21, WND or WNP as illustrated in the figure legends. After 24 and 48 h of co-incubation, used media were removed and cells were washed twice with PBS. Fresh medium containing 20 μl of MTT (5 mg/L) was added in and cells were cultured for an additional 4 h at 37°C in a 5% CO_2_incubator. After that, media were removed and 200 μl of DMSO was added in. The optical density value was determined by the microplate reader (Multiskan MK3, Thermo Fisher Scientific, Atlanta, GA, USA) at 492 nm.

### Cell cycle and apoptosis analyses

For cell cycle analysis by FACS, HepG2 cells were seeded in DMEM with 10% FBS in 6-well plates (1.5 ×10^5^ cells per well) and allowed to attach overnight. The medium was then changed to fresh DMEM with 10% FBS and cells were treated with PBS (control), WCN-21 (0.02 μM), WND (0.02 μM WCN-21) or WNP (0.02 μM WCN-21). Cells were trypsinized, collected and fixed in 70% ethanol at 4°C overnight. After being washed and resuspended in 200 μl PBS, cells were treated with 5 μl RNase (20 mg/ml) at 37°C for 30 min and stained with 20 μl propidium iodide (500 μg/ml, KeyGen Biotech Co. Ltd, Nanjing, China) at 4°C for 30 min. For apoptosis analysis, HepG2 cells were seeded in DMEM with 10% FBS in 6-well plates (1.5 × 10^5^ cells per well) and allowed to attach overnight. The medium was then changed to fresh DMEM with 10% FBS and cells were treated with PBS (control), WCN-21 (0.02 μM WCN-21), WND (0.02 μM WCN-21) or WNP (0.02 μM WCN-21) for 24 h. The cells were harvested by trypsinization and were stained using an Annexin V-FITC Apoptosis Detection Kit (KeyGen Biotech) according to the manufacturer’s protocol. Stained cells were immediately analyzed on a BD LSRFortessa X-20.

### Western blotting

HepG2 cells were incubated in CelLytic M Cell Lysis Reagent (Sigma-Aldrich, MA, USA) for 30 min on ice. The supernatant was collected after centrifugation at 12,000 rpm (Eppendorf 5415D). Cell lysates were separated on a 10% polyacrylamide gel and transferred to a PVDF membrane. The membrane was blocked for 1 h in 5% skim milk and then incubated with monoclonal antibody against cyclin A2 (1:1000, Abcam, Cambridge, UK), cyclin B1 (1:1000, Abcam), cyclin D1 (1:1000, Abcam), Bax (1:1000, Cell Signaling, Danvers, MA, USA), Bcl-2 (1:1000, Cell Signaling), caspase-3 (1:1000, Cell Signaling) or α-Tubulin (1:1000, Abcam) overnight. The membrane was washed in TBST (TBS with 0.1% Tween-20) three times and then incubated for 1 h with a secondary antibody. Then the membrane was washed four times and developed by an enhanced chemiluminescence system according to the manufacturer’s instructions (Perkin Elmer, Waltham, MA, USA).

### Pharmacokinetic analysis

Free WCN-21, WND or WNP were injected into Kunming mice as a single intravenous bolus via the lateral tail vein at a dose of 4 mg/kg WCN-21, 3 mice for each group. At 0 min, 15 min, 0.5 h, 1 h and 2 h after the injection, 500 μl blood was collected in heparin-treated tubes and then centrifuged at room temperature (Eppendorf 5415D, 5,000 rpm, 5 min) to obtain plasma. Plasma aliquots of 200 μl were added to 200 μl methyl alcohol and 400 μl DMSO. The samples were then vortexed for 30 s and centrifuged at 4°C (12000 × g, 10 min) to extract the WCN-21 from the plasma protein. WCN-21 concentration in plasma was measured based on its fluorescence intensity at 360 nm by fluorescence spectrophotometer (Hitachi F-4500, software FL solution, Japan). The concentration of WCN-21 in each sample was calculated by using a calibration curve determined by a series of dilution of WCN-21 solutions.

### Tissue distribution, tumor tissue uptake and anti-tumor efficacy study *in vivo*

For the tissue distribution, subcutaneous HepG2 tumors were seeded by inoculating 4 × 10^6^ HepG2 cells in the front armpit of the Balb/C nude mice. When tumor volume reach to ~50 mm^3^, Topotecan (4 mg/kg), free WCN-21 (4 mg/kg), WND (4 mg/kg WCN-21) or WNP (4 mg/kg WCN-21) was administrated by a single dose tail vein injection. Mouse lungs, hearts, livers, spleens, kidneys and tumor tissues were collected at 0, 1, 2 and 4 h after injection. Tissues of 0.1 g were homogenized with 500 μl saline and then centrifuged (Eppendorf 5415D, 12,000 rpm, 15 min) at 4°C to collect supernatant. The supernatant of 200 μl were added with 200 μl methyl alcohol and 400 μl DMSO. Then the mixture was then vortexed for 30 s and centrifuged at 4°C (12000 × g, 10 min) to extract obtain the WCN-21 supernatant from the tissue protein. Absorption of WCN-21 standard solutions and samples concentrations in tissues were measured with a fluorescence spectrophotometer at an excitation wavelength of 360 nm (Hitachi F-4500, software FL solution, Japan). The concentration of WCN-21 in each sample was calculated by using a standard curve determined by a series of dilution of WCN-21 solutions.

For the efficacy study, 4 × 10^6^ HepG2 cells were injected in the front armpit of the Balb/C nude mice. When tumors grew to ~50 mm^3^, free WCN-21, WND or WNP was injected via the lateral tail vein at a dose of 4 mg/kg WCN-21 every other day for total 5 doses. The tumor sizes were measured every other day and any death of the mice was recorded. Uptake of WCN-21 in tumor tissue was assayed using the tumor tissue resected from mice for distribution study. TUNEL, Ki67 and hematoxylin and eosin (H&E) staining were performed by using the resected tumor tissue from mice for efficacy study. The tissue sections were examined and photographed with a microscope (Olympus SZX12, Japan) connected to a PC.

### Statistical analysis

Comparison of two groups was performed using Student’s *t*-test (SPSS Software, Chicago, IL). Multiple groups were compared by one-way ANOVA with Dunnett’s post -test. A value of *p* < 0.05 was considered significant and *p* < 0.01 was considered highly significant.

## SUPPLEMENTARY MATERIALS FIGURES



## References

[1] Li QY, Zu YG, Shi RZ, Yao LP (2006). Review camptothecin: current perspectives. Current medicinal chemistry.

[2] Lorence A, Medina-Bolivar F, Nessler CL (2004). Camptothecin and 10-hydroxycamptothecin from Camptotheca acuminata hairy roots. Plant Cell Reports.

[3] Taschner-Mandl S, Schwarz M, Blaha J, Kauer M, Kromp F, Frank N, Rifatbegovic F, Weiss T, Ladenstein M, Hohenegger R, Ambros IM, Ambros PF (2016). Metronomic topotecan impedes tumor growth of MYCN-amplified neuroblastoma cells *in vitro* and *in vivo* by therapy induced senescence. Oncotarget.

[4] Zuco V, De Cesare M, Zaffaroni N, Lanzi C, Cassinelli G (2015). PLK1 is a critical determinant of tumor cell sensitivity to CPT11 and its inhibition enhances the drug antitumor efficacy in squamous cell carcinoma models sensitive and resistant to camptothecins. Oncotarget.

[5] Huang Q, Wang L, Lu W (2013). Evolution in medicinal chemistry of E-ring-modified Camptothecin analogs as anticancer agents. European journal of medicinal chemistry.

[6] Tukey RH, Strassburg CP, Mackenzie PI (2002). Pharmacogenomics of human UDP-glucuronosyltransferases and irinotecan toxicity. Mol Pharmacol.

[7] Rouits E, Charasson V, Petain A, Boisdron-Celle M, Delord JP, Fonck M, Laurand A, Poirier AL, Morel A, Chatelut E, Robert J, Gamelin E (2008). Pharmacokinetic and pharmacogenetic determinants of the activity and toxicity of irinotecan in metastatic colorectal cancer patients. Brit J Cancer.

[8] Bala V, Rao S, Boyd BJ, Prestidge CA (2013). Prodrug and nanomedicine approaches for the delivery of the camptothecin analogue SN38. Journal of controlled release.

[9] Huang Q, Wang L, Lu W (2013). Evolution in medicinal chemistry of E-ring-modified Camptothecin analogs as anticancer agents. European journal of medicinal chemistry.

[10] Jain RK, Stylianopoulos T (2010). Delivering nanomedicine to solid tumors. Nature reviews Clinical oncology.

[11] Liu Y, Miyoshi H, Nakamura M (2007). Nanomedicine for drug delivery and imaging: a promising avenue for cancer therapy and diagnosis using targeted functional nanoparticles. International journal of cancer.

[12] He Z, Huang J, Xu Y, Zhang X, Teng Y, Huang C, Wu Y, Zhang X, Zhang H, Sun W (2015). Co-delivery of cisplatin and paclitaxel by folic acid conjugated amphiphilic PEG-PLGA copolymer nanoparticles for the treatment of non-small lung cancer. Oncotarget.

[13] Yang T, Li B, Qi S, Liu Y, Gai Y, Ye P, Yang G, Zhang W, Zhang P, He X, Li W, Zhang Z, Xiang G (2014). Co-delivery of doxorubicin and Bmi1 siRNA by folate receptor targeted liposomes exhibits enhanced anti-tumor effects *in vitro* and *in vivo*. Theranostics.

[14] Yang T, Zhao P, Rong Z, Li B, Xue H, You J, He C, Li W, He X, Lee RJ, Ma X, Xiang G (2016). Anti-tumor Efficiency of Lipid-coated Cisplatin Nanoparticles Co-loaded with MicroRNA-375. Theranostics.

[15] Lu Y, Chen Y, Gemeinhart RA, Wu W, Li T (2015). Developing nanocrystals for cancer treatment. Nanomedicine.

[16] Müller RH, Gohla S, Keck CM (2011). State of the art of nanocrystals–special features, production, nanotoxicology aspects and intracellular delivery. European Journal of Pharmaceutics and Biopharmaceutics.

[17] Chen F, Zhao Y, Pan Y, Xue X, Zhang X, Kumar A, Liang XJ (2015). Synergistically Enhanced Therapeutic Effect of a Carrier-Free HCPT/DOX Nanodrug on Breast Cancer Cells through Improved Cellular Drug Accumulation. Mol Pharm.

[18] Zhou M, Zhang X, Yang Y, Liu Z, Tian B, Jie J, Zhang X (2013). Carrier-free functionalized multidrug nanorods for synergistic cancer therapy. Biomaterials.

[19] Zhao Y, Chen F, Pan Y, Li Z, Xue X, Okeke CI, Wang Y, Li C, Peng L, Wang PC, Ma X, Liang XJ (2015). Nanodrug Formed by Coassembly of Dual Anticancer Drugs to Inhibit Cancer Cell Drug Resistance. ACS applied materials & interfaces.

[20] Zhang J, Li Y, An FF, Zhang X, Chen X, Lee CS (2015). Preparation and size control of sub-100 nm pure nanodrugs. Nano letters.

[21] Nagarwal RC, Kumar R, Dhanawat M, Das N, Pandit JK (2011). Nanocrystal technology in the delivery of poorly soluble drugs: an overview. Current drug delivery.

[22] Chang TL, Zhan H, Liang D, Liang JF (2015). Nanocrystal technology for drug formulation and delivery. Frontiers of Chemical Science and Engineering.

[23] Peltonen L, Hirvonen J (2010). Pharmaceutical nanocrystals by nanomilling: critical process parameters, particle fracturing and stabilization methods. Journal of Pharmacy and Pharmacology.

[24] Patravale V, Kulkarni R (2004). Nanosuspensions: a promising drug delivery strategy. Journal of pharmacy and pharmacology.

[25] Gao L, Zhang D, Chen M (2008). Drug nanocrystals for the formulation of poorly soluble drugs and its application as a potential drug delivery system. Journal of Nanoparticle Research.

[26] Junghanns JUA, Müller RH (2008). Nanocrystal technology, drug delivery and clinical applications. International journal of nanomedicine.

[27] Li W, Zhang X, Hao X, Jie J, Tian B, Zhang X (2013). Shape design of high drug payload nanoparticles for more effective cancer therapy. Chemical communications.

[28] Chan HK, Kwok PCL (2011). Production methods for nanodrug particles using the bottom-up approach. Advanced drug delivery reviews.

[29] Zhang J, Li S, An FF, Liu J, Jin S, Zhang JC, Wang PC, Zhang X, Lee CS, Liang XJ (2015). Self-carried curcumin nanoparticles for *in vitro* and *in vivo* cancer therapy with real-time monitoring of drug release. Nanoscale.

[30] Dirksen JA, Ring TA (1991). Fundamentals of crystallization: Kinetic effects on particle size distributions and morphology. Chemical Engineering Science.

[31] Chen JF, Zhou MY, Shao L, Wang YY, Yun J, Chew NYK, Chan HK (2004). Feasibility of preparing nanodrugs by high-gravity reactive precipitation. International journal of pharmaceutics.

[32] Schwarzer HC, Schwertfirm F, Manhart M, Schmid HJ, Peukert W (2006). Predictive simulation of nanoparticle precipitation based on the population balance equation. Chemical Engineering Science.

[33] Wang Z, Chen JF, Le ZG, Shen Y, Yun J (2007). Preparation of Ultrafine Beclomethasone Dipropionate Drug Powder by Antisolvent Precipitation. Industrial & Engineering Chemistry Research.

[34] Chauhan VP, Popovic Z, Chen O, Cui J, Fukumura D, Bawendi MG, Jain RK (2011). Fluorescent nanorods and nanospheres for real-time *in vivo* probing of nanoparticle shape-dependent tumor penetration. Angewandte Chemie.

[35] Popović Z, Liu W, Chauhan VP, Lee J, Wong C, Greytak AB, Insin N, Nocera DG, Fukumura D, Jain RK, Bawendi MG (2010). A Nanoparticle Size Series for *In Vivo* Fluorescence Imaging. Angewandte Chemie.

[36] Gao L, Liu G, Ma J, Wang X, Zhou L, Li X (2012). Drug nanocrystals: *In vivo* performances. Journal of controlled release.

[37] Gao L, Zhang D, Chen M, Duan C, Dai W, Jia L, Zhao W (2008). Studies on pharmacokinetics and tissue distribution of oridonin nanosuspensions. International journal of pharmaceutics.

