# Supplementary material for: Nanocrystals of a new camptothecin derivative WCN-21 enhance its solubility and efficacy
Source: Oncotarget. 2017 Mar 13;8(18):29808–22. doi: 10.18632/oncotarget.16159 (PMC5444705; doi:10.18632/oncotarget.16159)
Supplement: Supplementary file 1 [file oncotarget-08-29808-s001.pdf]

# Nanocrystals of a new camptothecin derivative WCN-21 enhance its solubility and efficacy

## Supplementary Materials

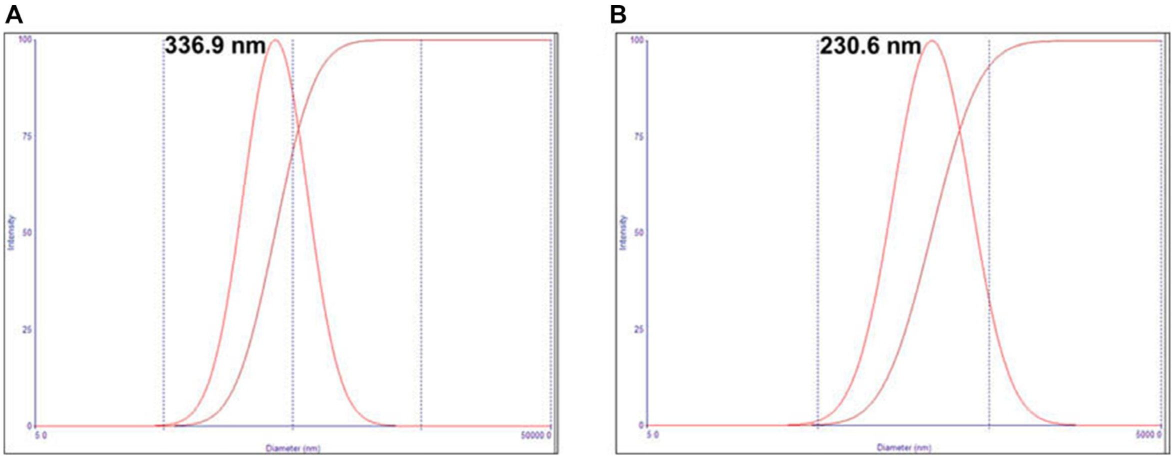


**Supplementary Figure 1: Size distribution of WND and WNP.** (**A**) WND size distribution, PDI = 0.304. (**B**) WNP size distribution, PDI = 0.258

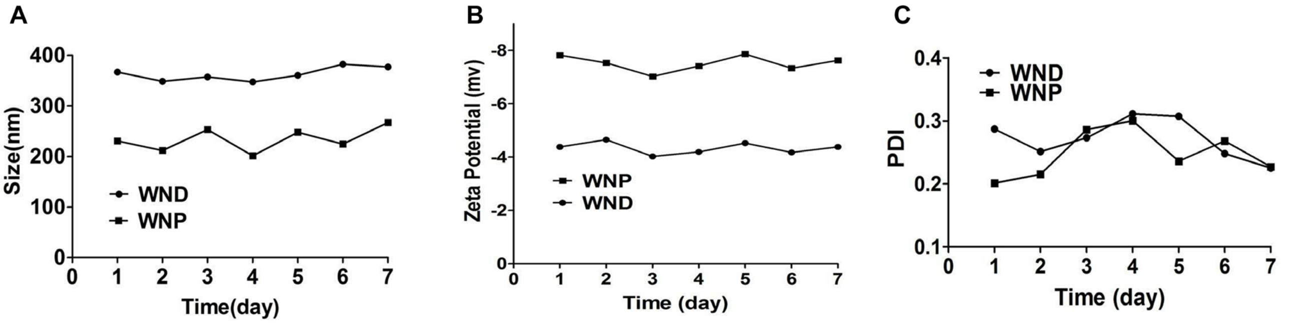


**Supplementary Figure 2: Stability of WND and WNP in aqueous solution for 7 days.** The size (**A**), zeta potential (**B**) and PDI (**C**) changes of WND and WNP were determined for 7 days.

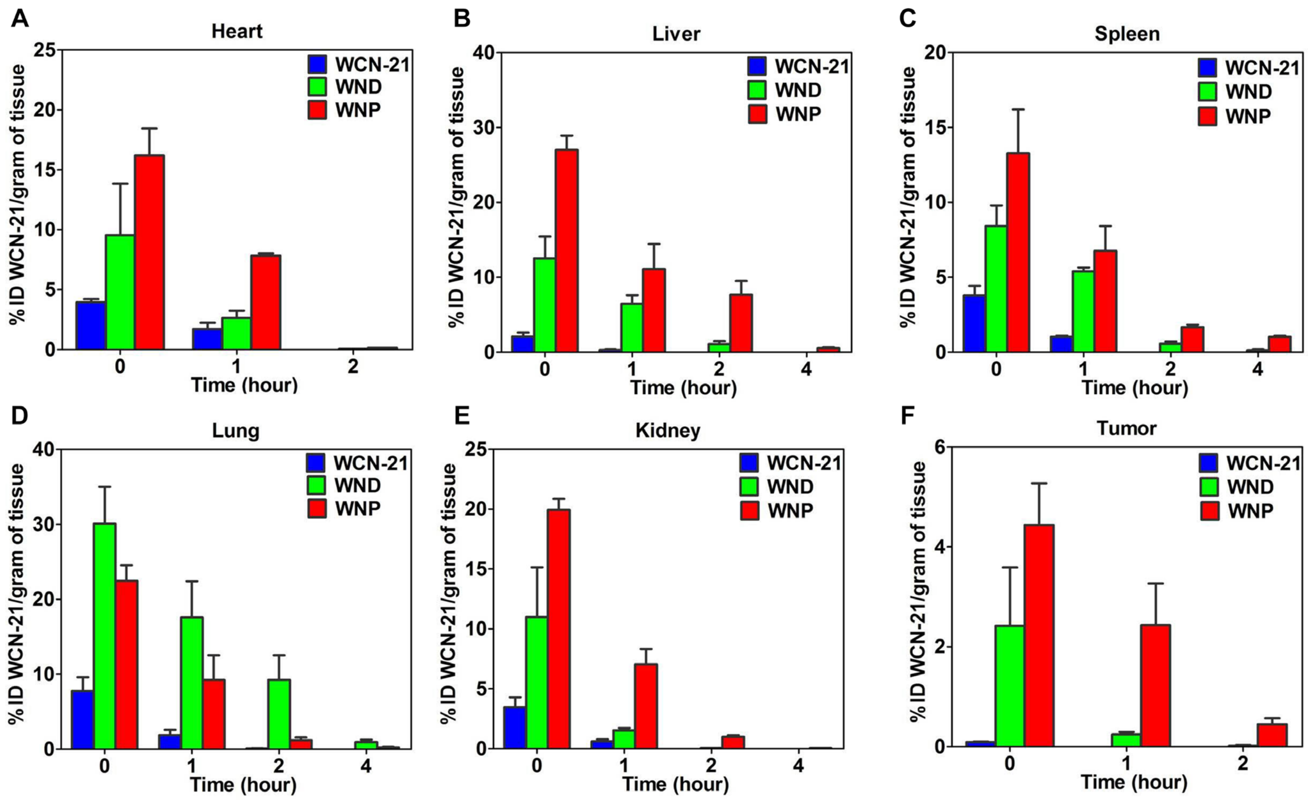


**Supplementary Figure 3: Biodistribution of WCN-21, WND, and WNP in xenograft mouse model.** Mice were injected (i.v.) with WCN-21 (4 mg/kg), WND (4 mg/kg WCN-21), or WNP (4 mg/kg WCN-21) and tissues were collected at 0, 1, 2, and 4 h post injection. Data are presented as average ± standard error ($n = 3$), and the statistical significance level is $^{**}p < 0.01$ and $^{***}p < 0.001$.
